# Comparison of Depolarization and Repolarization Parameters in Left vs. Right Ventricular Septal Pacing—An Intraprocedural Electrocardiographic Study

**DOI:** 10.3390/jcdd10030108

**Published:** 2023-03-04

**Authors:** Catalin Pestrea, Ecaterina Cicala, Madalina Ivascu, Alexandra Gherghina, Florin Ortan, Dana Pop

**Affiliations:** 1 Department of Interventional Cardiology, Brasov County Clinical Emergency Hospital, 500326 Brasov, Romania; 2 5th Department of Internal Medicine, Faculty of Medicine, “Iuliu Hațieganu” University of Medicine and Pharmacy, 400012 Cluj-Napoca, Romania; 3 Department of Cardiology, Clinical Rehabilitation Hospital, 400347 Cluj-Napoca, Romania

**Keywords:** left bundle branch area pacing, right ventricular septal pacing, electrocardiography, depolarization parameters, repolarization parameters

## Abstract

Compared with conventional right ventricular septal pacing (RVSP), several studies have shown a net clinical benefit of left bundle branch area pacing (LBBAP) in terms of ejection fraction preservation and reduced hospitalizations for heart failure. The purpose of this study was to compare acute depolarization and repolarization electrocardiographic parameters between LBBAP and RVSP in the same patients during the LBBAP implant procedure. We prospectively included 74 consecutive patients subjected to LBBAP from 1 January to 31 December 2021 at our institution in the study. After the lead was placed deep into the ventricular septum, unipolar pacing was performed and 12-lead ECGs were recorded from the distal (LBBAP) and proximal (RVSP) electrodes. QRS duration (QRSd), left ventricular activation time (LVAT), right ventricular activation time (RVAT), QT and JT intervals, QT dispersion (QTd), T-wave peak-to-end interval (Tpe), and Tpe/QT were measured for both instances. The final LBBAP threshold was a 0.7 ± 0.31 V at 0.4 ms duration with a sensing threshold of 10.7 ± 4.1 mV. RVSP produced a significantly larger QRS complex than the baseline QRS (194.88 ± 17.29 ms vs. 141.89 ± 35.41 ms, *p* < 0.001), while LBBAP did not significantly change the mean QRSd (148.10 ± 11.52 ms vs. 141.89 ± 35.41 ms, *p* = 0.135). LVAT (67.63 ± 8.79 ms vs. 95.89 ± 12.02 ms, *p* < 0.001) and RVAT (80.54 ± 10.94 ms vs. 98.99 ± 13.80 ms, *p* < 0.001) were significantly shorter with LBBAP than with RVSP. Moreover, all the repolarization parameters studied were significantly shorter in LBBAP than in RVSP (QT—425.95 ± 47.54 vs. 487.30 ± 52.32; JT—281.85 ± 53.66 vs. 297.69 ± 59.02; QTd—41.62 ± 20.07 vs. 58.38 ± 24.44; Tpe—67.03 ± 11.19 vs. 80.27 ± 10.72; and Tpe/QT—0.158 ± 0.028 vs. 0.165 ± 0.021, *p* < 0.05 for all), irrespective of the baseline QRS morphology. LBBAP was associated with significantly better acute depolarization and repolarization electrocardiographic parameters compared with RVSP.

## 1. Introduction

Physiological pacing has received significant interest in the last decade as a response to the growing evidence that conventional right ventricular pacing (RVP) is associated with a decrease in left ventricular function and heart failure, a condition termed pacing-induced cardiomyopathy [1]. Two cardiac pacing techniques have been implemented in routine clinical practice with the aim of capturing either the His bundle or the left bundle branch and, consequently, using the intrinsic conduction system for ventricular electrical activation. Left bundle branch area pacing (LBBAP) has gradually become the preferred option for physiological pacing in many laboratories due to a wider target area and better pacing and sensing thresholds compared with His bundle pacing [2]. Moreover, compared with conventional RVP, several studies have shown a net clinical benefit of LBBAP in terms of ejection fraction preservation and reduced hospitalizations for heart failure [3]. This benefit is explained in part by a narrower QRS complex as the result of faster ventricular depolarization.

The aspect and duration of the paced QRS complex have been topics of significant interest over the years. The left bundle branch block (LBBB)-like pattern induced by RVP triggers and maintains inter- and intraventricular electrical dyssynchrony, which is the basis for progressive ventricular mechanical dysfunction over time. Studies using activation mapping have shown that LBBB induced by RVP may worsen conduction barriers in the left ventricle compared with baseline LBBB [4]. Along with the pacing burden, the duration of the paced QRS complex is considered a major predictor of pacing-induced cardiomyopathy, with a directly proportional relationship between the QRS duration and the risk of left ventricular dysfunction occurrence [5]. Upgrading from RVP to LBBAP in patients with pacing-induced cardiomyopathy resulted in a significantly narrower paced QRS complex and significant improvements in left ventricular volumes and function [6]. Moreover, LBBAP showed better outcomes in patients with conventional indications for cardiac resynchronization therapy compared with biventricular pacing in terms of QRS narrowing and ejection fraction improvement [7].

From an electrocardiographic point of view, most studies have focused on and reported measurements of ventricular depolarization. Therefore, little data have been published on repolarization during physiological pacing [8]. Electrocardiographic parameters of repolarization, such as QT and JT intervals, QT dispersion (QTd), and T-wave peak-to-end interval (Tpe), have been studied as predictors of sudden cardiac death, both in the general population and in patients with implantable devices, especially defibrillators and biventricular pacemakers [9,10]. These studies have shown that increased values of these postprocedural parameters are associated with a higher risk of malignant ventricular arrhythmia occurrence due to the inhomogeneity of myocardial repolarization between different parts or layers of the myocardium creating the basis for arrhythmogenesis. Recent data have shown that LBBAP improved the dispersion of repolarization in heart failure patients, and this improvement was associated with positive echocardiographic response [11].

The hypothesis of this study was that LBBAP generates better electrocardiographic depolarization parameters (thus abbreviating known predictors for heart failure development and promoting left ventricular function preservation or improvement) and better repolarization parameters (thus reducing the risk of future malignant arrhythmias) compared with RVP.

In order to test this hypothesis, we compared acute depolarization and repolarization electrocardiographic parameters between left ventricular septal pacing and right ventricular septal pacing in the same patients during the LBBAP implant procedure.

## 2. Materials and Methods

### 2.1. Study Design

This was a prospective, analytical, single-center study.

### 2.2. Patient Selection

All consecutive patients who underwent LBBAP, either for bradyarrhythmias or for cardiac resynchronization therapy, between 1 January and 31 December 2021, in the Cardiac Pacing Laboratory of the Brașov County Clinical Emergency Hospital in Romania, were eligible for inclusion in the study. For the patients with symptomatic bradyarrhythmias, the main criterion for choosing LBBAP over another pacing strategy was an expected high ventricular pacing burden that would be encountered in different atrioventricular node disorders (e.g., atrioventricular block, slow-conducting atrial fibrillation, pace, and ablate strategy). Moreover, patients less than 75 years old and patients with a decreased baseline ejection fraction, but without criteria for resynchronization therapy, and with an indication for cardiac pacing, were considered for physiological pacing. The inclusion criteria were definite proof of lead penetration to the left side of the septum, the adequate capture of both left and right sides of the septum (see below), and twelve-lead electrocardiogram (12-lead ECG) recordings throughout the procedure. In the end, 74 patients were enrolled in the analysis.

The baseline demographic and clinical characteristics of the patients were recorded.

### 2.3. Pacing Procedure

The LBBAP procedure at our institution was performed as follows: either a Medtronic C315 His or C304 His catheter (Medtronic, Minneapolis, MN, USA) was first placed at the atrioventricular junction, a Medtronic Select Secure 3830 lead (Medtronic, Minneapolis, MN, USA) was used to record the His bundle electrogram, and the position of the His bundle was stored as a fluoroscopic reference. The catheter was then advanced in the right anterior oblique projection approximately 1.5–2 cm toward the apex and maintained perpendicular to the septum. The lead was advanced deep into the septum with clockwise rotations under continuous fluoroscopy monitoring in the left anterior oblique projection. Lead advancement was stopped when ventricular premature beats with a right bundle branch block morphology (RBBB) appeared on the 12-lead ECG. Successful LBBAP was defined according to the commonly used criteria in the literature as a paced QRS complex from the distal electrode of RBBB morphology and one of the following: a recorded left bundle branch potential, a left ventricular activation time less than 80 ms for baseline narrow QRS, and less than 90 ms for baseline wide QRS complex or proof of transition from nonselective to selective pacing with decreasing pacing amplitude [12].

**Figure 1:**
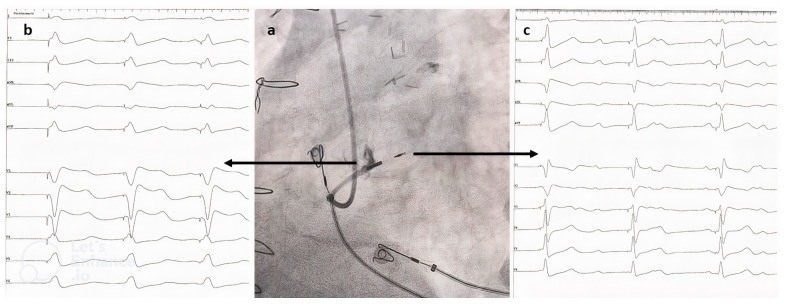
(**a**) Left anterior oblique fluoroscopic image showing the depth of lead penetration into the interventricular septum (with right septal delineation after contrast injection over the delivery catheter). The proximal electrode is in contact with the right side and the distal electrode is in contact with the left side of the septum. (**b**) Electrocardiogram of unipolar pacing from the proximal electrode to achieve RVSP. (**c**) Electrocardiogram of unipolar pacing from the distal electrode to achieve LBBAP. RVSP—right ventricular septal pacing; LBBAP—left bundle branch area pacing.

The depth of penetration was assessed with contrast injection delineating the right side of the septum, ensuring that the proximal electrode was in contact with the septum (Figure 1a).

Using the programmer, unipolar pacing at the same rate (20 bpm above the baseline rate) and the same amplitude (usually 2V at 0.4 ms) was performed at the distal electrode to capture the left side of the septum (termed LBBAP for the purpose of this study), and then at the proximal electrode to capture the right side of the septum (termed RVSP for the purpose of this study). We recorded the 12-lead ECGs for both settings after 30 s of pacing (Figure 1b,c). Additionally, final pacing and sensing thresholds, as well as procedural complications, total fluoroscopy, and procedural times, were noted.

### 2.4. Electrocardiographic Parameters

The 12-lead ECGs during the procedure were recorded using the Workmate Claris EP system (Abbott Cardiovascular, Plymouth, MN, USA). All electrocardiographic measurements were performed with the optimal augmentation at 50 mm/s sweep speed using the calipers provided by the system. The beginning of the QRS complex was considered the pacing artifact for RVSP and for nonselective LBBAP and the first deflection of the QRS complex in selective LBBAP when an isoelectric interval was recorded after the pacing artifact. The end of the T wave was determined using the tangent method (the intersection between the tangent to the final slope of the T wave and the isoelectric line). The leads without a clear T wave were excluded from the analysis. The following ECG measurements were performed for both RVSP and LBBAP:

- QRS duration (QRSd).
- Left ventricular activation time (LVAT)—during RVSP, from the first R wave notch in leads V5-V6 to the end of the QRS complex; during LBBAP, from the beginning of the QRS complex to the peak of R wave in leads V5-V6 [13].
- Right ventricular activation time (RVAT)—the difference between QRSd and LVAT [13].
- QT interval—longest QT interval measured in any lead.
- JT interval—the difference between QT and QRSd.
- QTd—the difference between the longest and shortest QT interval measured in any lead.
- Tpe—the mean of the intervals between the peak and the end of the T wave measured in all leads.
- Tpe/QT—the ratio between the Tpe and the QT interval.

### 2.5. Follow-Up

Patients were followed for a period of 12 months with pacing and sensing parameters, and complications were recorded at the end of the follow-up period.

### 2.6. Statistical Analysis

Continuous variables are presented as mean ± one standard deviation or as median and interquartile range. Categorical variables are presented as frequencies and percentages. A statistical comparison of means within the same group was performed using the *t*-test or Wilcoxon test for dependent groups and for different groups using the *t*-test or the Mann–Whitney U test for independent groups according to the normality of distribution. A confidence interval of 95% was used for all tests and a *p* < 0.05 was considered statistically significant.

Statistical analysis was performed using SPSS software v 26.0 (IBM, Armonk, NY, USA).

### 2.7. Ethical Considerations

This study complied with all aspects of the Declaration of Helsinki and was approved by the institutional ethics committee.

All patients were informed and provided their written consent before the procedure.

## 3. Results

### 3.1. Patient Characteristics

The baseline characteristics of the patients are presented in Table 1.

### 3.2. Procedural Characteristics

The final LBBAP threshold was 0.7 ± 0.31 V for a 0.4 ms pulse duration with a sensing threshold of 10.7 ± 4.1 mV and an impedance of 557.16 ± 158.5 Ohm. The fluoroscopy time was 10.44 ± 7.33 min, and the total procedural time was 118.36 ± 27.35 min. There were three intraprocedural septal perforations with lead migration into the left ventricular cavity managed with lead retraction and repositioning at another site, without further issues, and two cases of acute chest pain during lead fixation without signs of myocardial ischemia, which spontaneously resolved after the procedure.

The twelve-month follow-up showed an improved pacing threshold (0.58 ± 0.14 V at 0.4 ms pulse width, *p* = 0.014) and constant sensing parameters (10.62 ± 3.71 mV, *p*= 0.64). There was one case of lead dislodgement, without any other lead- or procedure-related complications.

### 3.3. Electrocardiographic Data

#### 3.3.1. Depolarization Parameters

**Figure 2:**
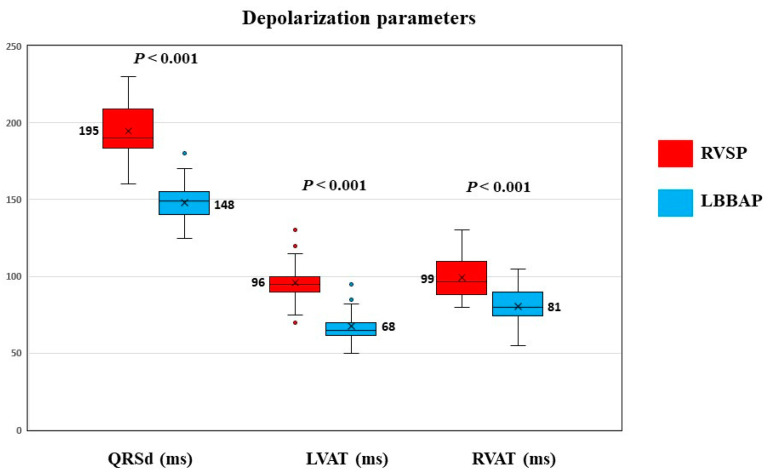
Comparison of depolarization electrocardiographic parameters between RVSP and LBBAP for the study group. RVSP—right ventricular septal pacing, LBBAP—left bundle branch area pacing, QRSd—QRS duration, LVAT—left ventricular activation time, RVAT—right ventricular activation time, red and blue dots – outlier points.

RVSP produced a significantly larger QRS complex than the baseline QRS (194.88 ± 17.29 ms vs. 141.89 ± 35.41 ms, *p* < 0.001), while LBBAP did not significantly change the mean QRS duration (148.10 ± 11.52 ms vs. 141.89 ± 35.41 ms, *p* = 0.135). LBBAP was associated with significantly improved depolarization parameters in the entire study group (Figure 2). The QRSd (148.10 ± 11.52 ms vs. 194.88 ± 17.29 ms, *p* < 0.001), LVAT (67.63 ± 8.79 ms vs. 95.89 ± 12.02 ms, *p* < 0.001), and RVAT (80.54 ± 10.94 ms vs. 98.99 ± 13.80 ms, *p* < 0.001) were significantly shorter with LBBAP than with RVSP.

RVSP generated significantly longer depolarization parameters in patients with baseline LBBB compared with those with baseline narrow QRS complex, while LBBAP produced similar depolarization parameters regardless of the baseline QRS morphology (Table 2).

#### 3.3.2. Repolarization Parameters

All the repolarization parameters studied were significantly shorter in LBBAP than in RVSP in the study group. The results are presented in Table 3.

As shown in Table 4, LBBAP was associated with non-statistically significant differences in repolarization parameters across all baseline QRS morphologies. The same result was recorded for RVSP.

## 4. Discussion

The main findings of this study were that LBBAP resulted in: (i) significantly better depolarization parameters, including shorter left and right ventricular activation times; and (ii) significantly better repolarization parameters, irrespective of baseline QRS morphology.

The observation that the paced QRS complex is narrower in LBBAP than in RVP is supported by several published studies [14]. The explanation resides in the direct capture of left bundle branch ramifications, which leads to rapid and synchronous left ventricular activation and, thus, a shorter LVAT. LVAT has been widely used as a proof and criterion for left bundle branch capture. Although a definite cut-off value with very high sensitivity and specificity is not known, the shorter the LVAT value, the higher the chance that the left bundle has been captured [15]. In our study, a mean value of LVAT during LBBAP lower than 70 ms suggested that the conduction system was engaged in the majority of cases.

On the other hand, the activation of the right ventricle in LBBAP is not completely understood. There are two possible mechanisms involved: transseptal myocardial activation from the tip of the lead and rapid activation through the conduction system (retrograde conduction up the left bundle and then down the right bundle) [15]. Regardless of the dominant mechanism, the advantage of LBBAP is that the activation of the left ventricle overlaps the activation of the right ventricle, resulting in a shorter RVAT when measured as the difference between the QRS duration and LVAT. In contrast, in RVSP, the ventricles are activated in sequence and through slow cell-to-cell conduction, generating longer right and left ventricular activation times and adding to a wider QRS complex.

Ventricular activation time is directly proportional to the conduction properties of the ipsilateral bundle. This explains why, irrespective of the baseline QRS complex (and left bundle conduction), the capture of the left bundle during LBBAP resulted in nonstatistically different LVATs. On the contrary, an interesting finding in our study was that RVAT was similar when LBBAP was performed in patients with baseline normal QRS and in patients with RBBB. Because the right bundle was slowly conducting or nonconducting in the latter patients at baseline, plausible explanations for this result could be the dominance of transseptal activation of the right ventricle, or recruitment, with a higher voltage, of dormant right bundle branch fibers during physiological pacing.

The limited existing data showed better repolarization parameters in LBBAP patients than in RVSP patients [8]. Although the QT interval is expected to be lower in LBBAP primarily due to a significantly shorter QRS duration, in our study, the JT interval was also significantly reduced, proving that the total repolarization duration is favorably influenced by the procedure.

Several studies have questioned the relevance of QTd as a measure of regional repolarization heterogeneity [16]. We think that the values identified in our study with LBBAP were a direct consequence of the rapid and synchronous activation of the left ventricle, which, in turn, caused different parts of the ventricle to start the repolarization process within a much narrower time interval. Furthermore, the values we recorded fall into the normal values described in large population studies (30–60 ms) [17]. In contrast, in RVSP, repolarization follows the sequence of depolarization, and some parts of the ventricle have a delayed recovery, leading to a wider dispersion.

Tp-Te and Tp-Te/QT are considered to reflect the last part of repolarization, are measures of transmural dispersion of repolarization in the left ventricle, and have been shown to predict ventricular arrhythmias [18]. Previous studies proved a significant increase in these parameters with both right ventricular endocardial and left ventricular epicardial pacing [19]. The significantly lower intervals noted with LBBAP than with RVSP in the present study most likely resulted from the initial depolarization that occurs through the native conduction system, leading to a similar endocardial-to-epicardial activation of the myocardial layers, as encountered during baseline narrow QRS rhythm.

The final LBBAP procedural parameters were similar to those described in large registries from experienced centers. Moreover, in line with the existing data, the one-year follow-up showed constant pacing and sensing thresholds and no major postprocedural complications, except one lead dislodgement, which needed reintervention [20].

One of the limitations of the study is the measurement of ventricular depolarization and repolarization times on the surface ECG, which may have led to less accurate values. Other techniques, such as noninvasive electrocardiographic mapping or invasive activation mapping, provide more accurate results but are more difficult to use intraoperatively. Nevertheless, reports using the latter techniques support our findings that LBBAP leads to rapid and synchronous ventricular activation [21].

Another limitation of the study derives from the difficulty with surface ECG interval measurements, especially the correct identification of the J point and the T-wave offset. Nevertheless, because this was an intrapatient study, and the measurements were made using the same tools, the measurement and selection biases associated with comparing different groups of patients were minimized and likely had no impact on the final results. This includes differences in intervals due to baseline structural or electrical heart diseases or an increased ventricular mass [22].

This was an acute intraprocedural study, and there is the possibility that the measured parameters could change over time. Unfortunately, once the permanent pacemaker is implanted, there is no possibility of pacing on the proximal electrode, so the reproduction of the study during follow-up, until the time for the box change, is impossible.

An important issue with studies that include patients with RVSP is that the final position of the lead with fluoroscopic guidance may not be true septal, as previously proved with postprocedural echocardiography or computer tomography imaging [23]. The strength of our study was that pacing was performed without a doubt on opposite parts of the septum, thus excluding the possibility of misplaced leads labeled as right septal pacing.

To the best of our knowledge, this is the first study to compare the LBBAP and RVSP electrocardiographic parameters of depolarization and repolarization in an intraindividual setting. Previous studies also reported electrocardiographic differences related to different pacing modalities, but the study populations consisted of separate study groups for each pacing strategy, and they did not investigate both repolarization and depolarization parameters (including both left and right ventricular activation times) [24]. The major advantage of our study was that the comparison between LBBAP and RVSP was performed at the same moment in time, in the same overall clinical setting, and during the same autonomous nervous system status. Furthermore, the strength of comparison was not affected by the underlying electrophysiologic properties of the conduction system or by antiarrhythmic medication, which may affect electrical conduction.

## 5. Conclusions

Left bundle branch area pacing is associated with significantly better acute depolarization and repolarization electrocardiographic parameters compared with right ventricular septal pacing.

## Figures and Tables

**Table 1:** Baseline patient characteristics.

N	74
Age (years, median [IQR])	71 [14]
Male sex	55 (74.32%)
BMI (kg/cm^2^, mean ± SD)	28.02 ± 4.60
Pacing indication	
Sick sinus node disease	4 (5.40%)
Atrioventricular block	34 (45.94%)
Resynchronization therapy	20 (27.02%)
Pace and ablate	6 (8.10%)
Pacing-induced cardiomyopathy	2 (2.70%)
Slow-conducting AF	8 (10.81%)
QRS duration (ms, mean ± SD)	143.04 ± 35.72
Normal QRS	38 (51.4%)
Left bundle branch block	24 (32.4%)
Right bundle branch block	12 (16.2%)
Comorbidities	
Atrial fibrillation	28 (37.83%)
Stroke	4 (5.40%)
Diabetes mellitus	25 (33.78%)
Hypertension	60 (81.08%)
Ischemic cardiomyopathy	26 (35.13%)
Renal insufficiency	21 (28.38%)
Obstructive lung disease	3 (4.05%)
Medical treatment	
Beta-blockers	64 (86.48%)
Angiotensin-converting enzyme inhibitors	52 (70.27%)
Mineralocorticoid receptor antagonists	40 (54.05%)
ARNI	18 (24.32%)
SGLT2 inhibitors	9 (12.16%)
Anticoagulants	32 (43.24%)

IQR—interquartile range, BMI—body mass index, SD—standard deviation, AF—atrial fibrillation, ARNI—angiotensin receptor—neprilysin inhibitor, and SGLT2—sodium-glucose cotransporter 2.

**Table 2:** Depolarization parameters according to baseline QRS morphology.

Parameters during RVSP	Baseline			*p* Value		
	Narrow QRS	LBBB	RBBB	Narrow vs. LBBB	Narrow vs. RBBB	LBBB vs. RBBB
QRS duration (ms, mean ± SD)	189.14 ± 13.77	204.57 ± 21.03	194 ± 10.79	0.004	0.271	0.058
LVAT (ms, mean ± SD)	93.03 ± 10.33	100.74 ± 14.24	95.42 ± 10.10	0.018	0.487	0.259
RVAT (ms, mean ± SD)	96.11 ± 13.13	103.83 ± 15.62	98.58 ± 9.97	0.044	0.553	0.301
Parameters during LBBAP	Baseline			*p* value		
	Narrow QRS	LBBB	RBBB	Narrow vs. LBBB	Narrow vs. RBBB	LBBB vs. RBBB
QRS duration (ms, mean ± SD)	146.41 ± 11.71	151.57 ± 11.98	146.67 ± 9.13	0.106	0.944	0.225
LVAT (ms, mean ± SD)	66.92 ± 8.08	68.13 ± 9.29	68.83 ± 10.44	0.596	0.511	0.840
RVAT (ms, mean ± SD)	79.62 ± 11.11	83.43 ± 11.02	77.83 ± 9.86	0.200	0.622	0.149

RVSP—right ventricular septal pacing, LBBAP—left bundle branch area pacing, LBBB—left bundle branch block, RBBB—right bundle branch block, LVAT—left ventricular activation time, RVAT—right ventricular activation time, and SD—standard deviation.

**Table 3:** Repolarization parameters in RVSP and LBBAP for the entire study group.

Parameter	RVSP	LBBAP	*p* Value
QT interval (ms, mean ± SD)	487.30 ± 52.32	425.95 ± 47.54	<0.001
JT interval (ms, mean ± SD)	297.69 ± 59.02	281.85 ± 53.66	<0.001
QT dispersion (ms, mean ± SD)	58.38 ± 24.44	41.62 ± 20.07	<0.001
T-peak end (ms, mean ± SD)	80.27 ± 10.72	67.03 ± 11.19	<0.001
T-peak end/QT (mean ± SD)	0.165 ± 0.021	0.158 ± 0.028	0.018

RVSP—right ventricular septal pacing, LBBAP—left bundle branch area pacing, and SD—standard deviation.

**Table 4:** Repolarization parameters according to baseline QRS morphology.

Parameters during RVSP	Baseline			*p* Value		
	Narrow QRS	LBBB	RBBB	Narrow vs LBBB	Narrow vs. RBBB	LBBB vs. RBBB
QT interval (ms, mean ± SD)	482.11 ± 53.33	495.83 ± 49.68	486.67 ± 56.46	0.315	0.800	0.621
JT interval (ms, mean ± SD)	297.95 ± 64.41	299.79 ± 55.45	292.67 ± 51.93	0.908	0.797	0.713
QT dispersion (ms, mean ± SD)	56.58 ± 26.02	62.92 ± 21.96	55.00 ± 24.68	0.326	0.854	0.335
Tpeak-end (ms, mean ± SD)	79.21 ± 10.75	82.50 ± 10.73	79.17 ± 10.83	0.245	0.990	0.387
Tpeak-end/QT (mean ± SD)	0.165 ± 0.020	0.166 ± 0.017	0.164 ± 0.029	0.746	0.953	0.788
Parameters during LBBAP	Baseline			*p* value		
	Narrow QRS	LBBB	RBBB	Narrow vs. LBBB	Narrow vs. RBBB	LBBB vs. RBBB
QT interval (ms, mean ± SD)	424.21 ± 51.44	428.33 ± 45.07	426.67 ± 42.71	0.749	0.882	0.916
JT interval (ms, mean ± SD)	281.66 ± 60.95	283.08 ± 48.73	280.00 ± 40.51	0.923	0.930	0.852
QT dispersion (ms, mean ± SD)	38.42 ± 19.24	44.58 ± 19.99	45.83 ± 22.74	0.231	0.271	0.867
Tpeak-end (ms, mean ± SD)	65.53 ± 11.07	69.58 ± 11.22	66.67 ± 11.54	0.167	0.760	0.471
Tpeak-end/QT (mean ± SD)	0.156 ± 0.028	0.163 ± 0.025	0.158 ± 0.035	0.304	0.796	0.644

RVSP—right ventricular septal pacing, LBBAP—left bundle branch area pacing, LBBB—left bundle branch block, RBBB—right bundle branch block, and SD—standard deviation.

## Data Availability

The datasets are available upon reasonable request to the corresponding author.

## Abbreviations

RVP—right ventricular pacing; LBBAP—left bundle branch area pacing; LBBB—left bundle branch block; QTd—QT dispersion; Tpe—T-wave peak-to-end interval; Tpe/QT—the ratio between the Tpe and the QT interval; RBBB—right bundle branch block; RVSP—right ventricular septal pacing; QRSd—QRS duration; LVAT—left ventricular activation time; RVAT—right ventricular activation time; IQR—interquartile rang; BMI—body mass index; SD—standard deviation; AF—atrial fibrillation; ARNI—angiotensin receptor—neprilysin inhibitor; SGLT2—sodium-glucose cotransporter 2.
